# Apolipoprotein B and Glycemic Dysregulation: New Predictors of Type 2 Diabetes in High-Cardiovascular-Risk Populations

**DOI:** 10.3390/jpm15050163

**Published:** 2025-04-23

**Authors:** Makhabbat Bekbossynova, Tatyana Ivanova-Razumova, Aknur Kali, Aliya Sailybayeva, Sadyk Khamitov, Gulnur Daniyarova, Kamila Akzholova, Timur Saliev

**Affiliations:** 1 Heart Center, Corporate Fund “University Medical Center”, Nazarbayev University, Astana 010000, Kazakhstan; m.bekbosynova@umc.org.kz (M.B.);; 2 School of Medicine, Nazarbayev University, Astana 010000, Kazakhstan; 3 Institute of Fundamental and Applied Medical Research, S.D. Asfendiyarov Kazakh National Medical University, Almaty 050000, Kazakhstan

**Keywords:** cardiovascular risk, lipid profile, glycemic profile, apolipoprotein B, lipoprotein(a), type 2 diabetes mellitus

## Abstract

**Background:** Apolipoprotein B (ApoB), a key component of atherogenic lipoproteins, has been increasingly implicated in cardiometabolic disorders beyond dyslipidemia. However, its role in glycemic dysregulation remains unclear. This study aimed to investigate the association between ApoB levels and glycemic parameters, including fasting glucose, insulin resistance, and glycated hemoglobin (HbA1c), in individuals without diagnosed diabetes. **Methods:** This study was conducted at the National Research Cardiac Surgery Center (Kazakhstan) over the period between 2023 and 2024 as a cross-sectional analysis. Adults aged ≥ 20 years without diagnosed diabetes and with complete data on their ApoB and glycemic markers were included. Associations between ApoB and fasting plasma glucose (FPG), HbA1c, and HOMA-IR were assessed using multivariable linear and logistic regression models adjusted for demographic, lifestyle, and metabolic covariates. **Results:** Higher ApoB levels were significantly associated with increased fasting glucose (β = 2.07 mg/dL per 1-SD increase in ApoB, *p* < 0.001), higher HbA1c (β = 0.06%, *p* < 0.001), and elevated HOMA-IR (β = 0.54, *p* < 0.001). Participants in the highest ApoB quartile had 53% higher odds of prediabetes (adjusted OR = 1.53; 95% CI: 1.22–1.91; *p* < 0.001) compared to the lowest quartile. These associations remained significant after adjusting for BMI, lipid levels, and other confounders. **Conclusions:** Elevated ApoB is independently associated with adverse glycemic profiles in nondiabetic individuals, suggesting its potential role in early glucose metabolism disturbances.

## 1. Introduction

Cardiovascular diseases (CVDs) remain the leading cause of morbidity and mortality worldwide, accounting for approximately 17.9 million deaths annually [1]. Among these, atherosclerotic cardiovascular diseases (ASCVDs), characterized by the build-up of plaque within the arterial walls, pose a significant health challenge [2]. The risk of developing an ASCVD is influenced by a complex interplay of metabolic factors, including dyslipidemia and impaired glucose metabolism [3,4]. These risk factors not only contribute to the onset of cardiovascular events but also exacerbate the clinical outcomes in high-risk populations.

Dyslipidemia, marked by abnormalities in lipid profiles such as elevated low-density lipoprotein cholesterol (LDL-C), high triglycerides, and low high-density lipoprotein cholesterol (HDL-C), is a well-established contributor to atherosclerosis [5,6]. Lipoprotein(a) [Lp(a)], a genetically inherited lipoprotein, has recently gained attention as an independent risk factor for cardiovascular events [7,8]. Elevated Lp(a) levels are associated with an increased risk of coronary artery disease, stroke, and peripheral arterial disease [9]. However, the relationship between Lp(a) and glucose metabolism, particularly type 2 diabetes mellitus (T2DM), remains complex and not fully elucidated [10,11,12]. While early studies suggested an inverse association between Lp(a) levels and the risk of T2DM [13], more recent data indicate a potential positive correlation between high Lp(a) levels and cardiovascular complications in diabetic patients [14,15]. This discrepancy underscores the need for further research into the dual impact of Lp(a) on cardiovascular and metabolic health [16].

Beyond Lp(a), other lipid parameters, including apolipoprotein B (Apo-B), apolipoprotein A (Apo-A), and triglycerides, play significant roles in cardiovascular risk assessment [17,18]. Apo-B, in particular, is a key marker of atherogenic lipoproteins, and its elevated levels have been associated with an increased risk of ASCVD and poor glycemic control [19,20]. The link between Apo-B and insulin resistance suggests that it may serve as a critical mediator between lipid metabolism and glucose homeostasis [21,22]. Similarly, disturbances in other lipid components can influence inflammatory pathways and endothelial function, further linking dyslipidemia with the pathogenesis of T2DM [16].

Kazakhstan, with its diverse population and unique epidemiological landscape, presents a critical setting for examining the relationship between lipid and glycemic profiles in very high-cardiovascular-risk groups [23,24]. Despite improvements in healthcare infrastructure, the country continues to experience high prevalence rates of ASCVD and diabetes, influenced by genetic predispositions, lifestyle factors, and healthcare access disparities [25,26]. The majority of the existing studies in Kazakhstan have focused on general population trends, leaving a gap in understanding the specific needs of high-risk patients who might benefit from tailored therapeutic interventions.

The objective of this study is to assess the relationship between lipid and glycemic profile parameters in patients with a very high cardiovascular risk in Kazakhstan. By identifying key predictors of T2DM and exploring how lipid abnormalities correlate with glucose metabolism, this research aims to enhance risk stratification and guide clinical decision-making.

This study also seeks to provide insights into potential therapeutic targets that could help mitigate cardiovascular risks and improve metabolic control in this vulnerable population.

## 2. Materials and Methods

**Figure 1:**
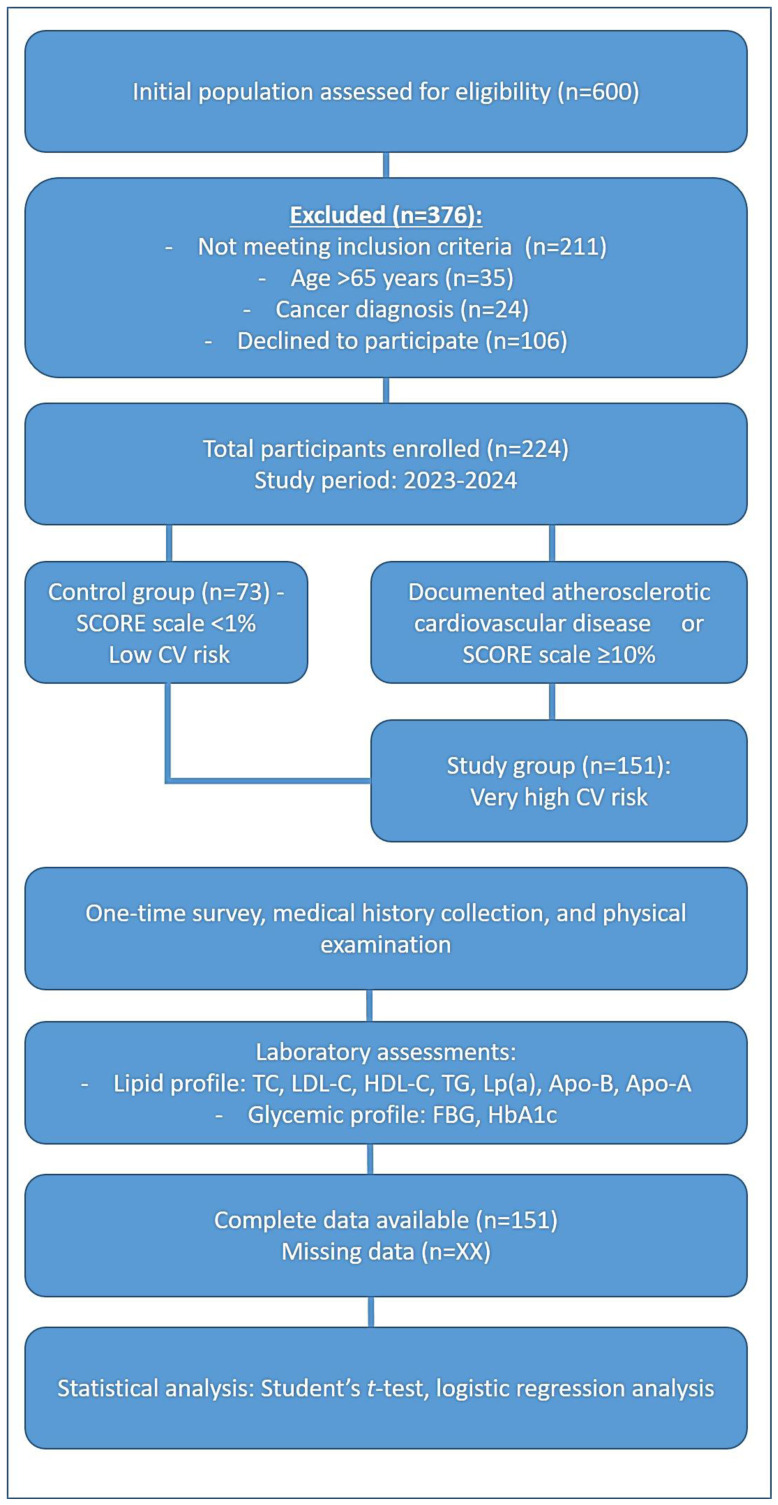
The scheme of the methodology of the clinical trial.

The study design included two groups, as illustrated in Figure 1. The study group comprised 151 patients classified as having very high cardiovascular risk, while the control group consisted of 73 individuals with low cardiovascular risk.

The inclusion criteria for the very high-risk group required the presence of at least one of the following factors: documented atherosclerotic cardiovascular disease (ASCVD) confirmed clinically or through imaging. This included a history of acute coronary syndrome (ACS) (myocardial infarction or unstable angina), stable angina, prior coronary revascularization procedures (percutaneous coronary intervention or coronary artery bypass grafting), stroke or transient ischemic attack (TIA), or evidence of significant coronary artery stenosis (atherosclerotic plaques) detected via coronary angiography or CT angiography. Patients with multiple coronary artery lesions or a 10-year risk of fatal cardiovascular events ≥ 10%, based on the SCORE (Systematic Coronary Risk Estimation) scale, were also included.

The exclusion criteria encompassed patients diagnosed with cancer and individuals over 65 years of age to maintain a more homogenous study population. Figure 1 represents the design of this clinical study, outlining the division of its participants into the study group (patients with very high cardiovascular risk) and the control group (healthy individuals). It highlights the inclusion and exclusion criteria used for participant selection.

Patients in the low-risk group were those with a SCORE scale < 1%, indicating a 10-year risk of fatal cardiovascular disease (CVD). This study adhered to the European Atherosclerosis Society (EAS) guidelines, which provided standardized criteria for classifying the individuals into the very high-risk and low-risk categories based on their cardiovascular profiles.

This study was conducted at the National Research Cardiac Surgery Center (Kazakhstan) over the period between 2023 and 2024 as a cross-sectional analysis. Each participant underwent a one-time assessment, including a comprehensive survey, a detailed medical history collection, and a physical examination.

Laboratory evaluations included both lipid and glycemic profile assessments. The lipid profile consisted of total cholesterol, LDL cholesterol, HDL cholesterol, lipoprotein(a) [Lp(a)], apolipoprotein B (Apo-B), and apolipoprotein A (Apo-A) (Table 1). The glycemic profile was assessed through fasting blood glucose levels and glycated hemoglobin (HbA1c) measurements (Table 2).

### 2.1. Patient and Public Involvement

Patients were recruited based on the inclusion criteria and by signing their informed consent. All interventions were conducted according to the internal protocols and requirements of the Ministry of Health, Kazakhstan. All methods of intervention were conducted after taking informed consent from the patients. The assessment of the burden of intervention was a mandatory requirement according to the Organization’s rules. The results of the clinical trial will be available to the participants and relevant wider patient communities through publications in journals and on the website of the Organization, where they are able to monitor the study’s progress. Also, annual reports will be submitted to the local Bioethics Committee with all the results of the study.

### 2.2. Sample Collection

Blood samples were collected from the patients upon hospital admission at the Heart Center, Corporate Fund “University Medical Center”, using standard venous phlebotomy techniques, placed into appropriate vacutainers (EDTA for CBC, SST for biochemical and lipid analysis, citrate for coagulation studies), processed via centrifugation at 3000 rpm for 10–15 min, stored at −20 °C to −80 °C as needed, and analyzed using automated hematology, chemistry, and coagulation analyzers. Patients underwent a complete clinical examination and first-level blood work-up, including Complete Blood Count (complete blood count using an analyzer with 5-class cell differentiation and neutrophil-to-lymphocyte ratio), Biochemical Blood Test (ALT, AST, total bilirubin, direct bilirubin, high-sensitivity CRP, creatinine, urea, glucose, glycated hemoglobin, D-dimer, and fibrinogen), Lipid Profile (LDLs, triglycerides, high-density lipoproteins (HDL), very low-density lipoproteins (VLDLs), ApoA, ApoB, and Lp(a)), Coagulogram (ferritin, iron, and hepatitis markers), and Other Blood Tests (NT-pro BNP, homocysteine, IL-6, galectin-3, troponin, and blood group determination).

### 2.3. Ethical Issues

The part of this study involving humans was approved by the local Bioethics Committee of the “University Medical Center” corporate fund (Heart Center, Corporate Fund University Medical Center, Nazarbayev University, Astana, Kazakhstan). Approval of the application for study No. 3/2023/PE was dated 14 July 2023. This study was conducted in accordance with the local legislation and institutional requirements. The participants provided their written informed consent to participate in this study.

### 2.4. Statistics

The statistical analysis included descriptive and analytical statistics. For variables with a normal distribution, parametric statistical methods were used for comparative analysis. For the analysis of variables with a normal data distribution, parametric statistical methods were used for comparative analysis. Numerical variables were presented as mean values ± standard deviation. To determine the degree of correlation between indicators, a correlation analysis was conducted, including the calculation of the correlation coefficient. The statistical significance of the comparisons was assessed using Student’s *t*-test for continuous variables that met the assumptions of normality and homogeneity of variance. For variables that did not satisfy these assumptions, appropriate non-parametric alternatives were applied.

To evaluate predictors of diabetes in patients with very high cardiovascular risk, a logistic regression analysis was conducted within this subgroup (*n* = 151). In the univariate models, crude odds ratios (ORs) with 95% confidence intervals (CIs) were calculated separately for each of the following independent variables: age, body mass index (BMI), low-density lipoprotein cholesterol (LDL-C), and apolipoprotein B (ApoB). These variables were then entered together into a multivariate logistic regression model to obtain adjusted ORs. To assess the robustness of the association between ApoB and diabetes, additional sensitivity analyses were performed.

Statistical analyses were performed using Python (version 3.11.11), SciPy library (version 1.14.1), and Statsmodels (version 0.14.4). Data visualization was conducted with the Matplotlib (version 3.10.0) and Seaborn (0.13.2) libraries.

## 3. Results

### 3.1. Baseline Characteristics

The main characteristics of the 224 study participants are presented in Table 3. Gender distribution: males constituted 70.8% of the study group and 40.8% of the control group, respectively. The average age in the very high-risk group was 58 ± 6.7 years, while in the control group it was 48 ± 10.4 years. Ethnic composition of the study groups: in the control group, Kazakh individuals made up 92%, while in the very high-risk group, they accounted for 81.05%. Table 3 shows the baseline characteristics of the participants in the control and study groups.

**Figure 2:**
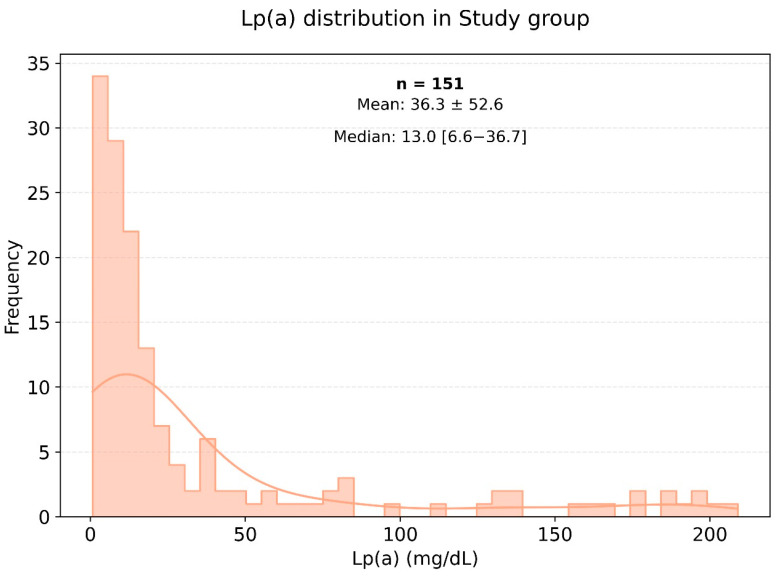
Frequency of occurrence of various levels of Lp(a) in a small group of very high-risk patients.

The average weight and body mass index (BMI) were significantly higher in the very high-risk cardiovascular disease group compared to the control group, with *p*-values of 0.001 and 0.003, respectively. Figure 2 illustrates the distribution of lipoprotein (a) [Lp(a)] levels among patients in the very high-cardiovascular-risk group, showing most commonly Lp(a) levels within the range of 0 to 25 mg/dL. In the very high-risk cardiovascular disease group, the median level of Lp(a) was 14.27 mg/dL, while in the control group, it was 10.4 mg/dL.

**Figure 3:**
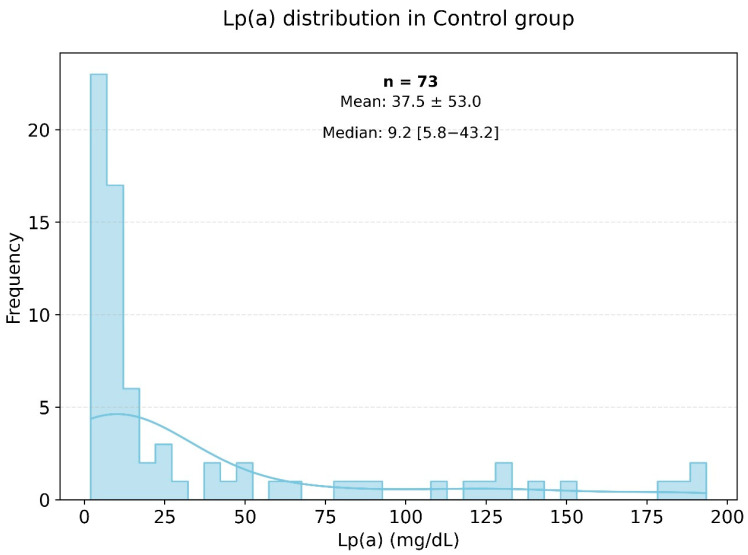
Frequency of occurrence of various levels of Lp(a) in the small control group.

Lipid profile analysis in the two groups showed pronounced statistically significant differences, as presented in Figure 2 and Figure 3. Specifically, in the study group, the triglyceride level was 155.18 ± 80.02 mmol/L, while in the control group, it was significantly lower at 120.94 ± 76.17 mmol/L (*p* < 0.003). The level of HDL cholesterol in the control group was significantly higher than in the very high-risk patients, with values of 55.60 ± 14.60 and 45.52 ± 11.74 mmol/L, respectively (*p* < 0.001). The levels of Apo-A were also significantly different, with the control group showing a higher level of 1.44 ± 0.26 g/L (*p* < 0.001). The Figure 3 histogram depicts the distribution of Lp(a) levels in the control group (healthy individuals), highlighting that most Lp(a) levels also fell within the range of 0 to 25 mg/dL.

### 3.2. Analysis of Lp(A) and Apo-B Levels

**Figure 4:**
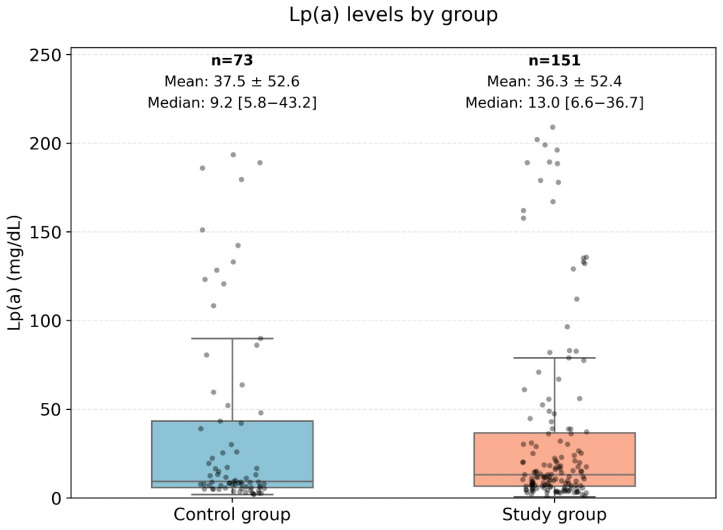
Comparison of ApoB levels between the control group and the very high-risk patient group. This Figure displays a boxplot comparison of the Apolipoprotein B (ApoB) levels between two groups: the control group (low-risk patients) and the high-risk group (very high-risk patients). The central box represents the interquartile range (IQR) where 50% of the data points are located, while the line inside the box represents the median ApoB level. The whiskers extend to the minimum and maximum values, excluding outliers, which are represented as individual points. This analysis used the Mann–Whitney U test to assess differences between the groups, with no statistically significant difference observed (U-statistic: 5798.0, *p*-value: 0.83).

The levels of Lp(a) and Apo-B in the presented data do not follow a normal distribution; therefore, non-parametric methods were used for the analysis. The data are presented in Figure 4.

In the very high-risk cardiovascular disease group, the median level of Lp(a) was 14.27 mg/dL, while in the control group, it was 10.4 mg/dL.

Figure 2 and Figure 3 show histograms of the distribution of Lp(a) levels based on the frequency of values in the very high-risk group. According to the presented data, the most common Lp(a) levels were in the range of 0 to 25 mg/dL, including among patients in the very high-risk group.

Figure 4 represents a comparison diagram of the Apo-B levels between low-risk patients (control group) and high-risk patients. The Mann–Whitney U test was applied to compare the ranks of the ApoB levels (U-statistic: 5798.0, *p*-value: 0.83). Therefore, no statistically significant differences in ApoB levels were found between patients with low and high cardiovascular risk.

### 3.3. Relationship Between Lipid and Glycemic Profile Indicators

In the very high-risk cardiovascular events group, type 2 diabetes was identified in 51 individuals, accounting for 20.5%, while 18 individuals (10.9%) had impaired glucose tolerance. In the control group, two cases of type 2 diabetes were diagnosed, representing 2.8%, and there was one case of impaired glucose tolerance (1.4%).

A correlation analysis of lipid profile indicators and glycemic parameters yielded the following results: No direct or inverse correlation was found between Lp(a) levels and fasting glucose levels or glycated hemoglobin (the correlation coefficients were −0.07 and −0.06, respectively). The correlation coefficient between Apo-B levels and glucose levels was 0.21, and between Apo-B levels and glycated hemoglobin was 0.24, indicating a weak positive relationship. Additionally, a weak negative relationship was found between Apo-A levels and glycemic profile parameters: the correlation coefficient with fasting glucose levels was −0.10, and with glycated hemoglobin levels was −0.24. The most pronounced positive relationship was observed between triglyceride levels and glycemic profile indicators, with correlation coefficients of 0.23 and 0.32, respectively.

### 3.4. Lipid Profile and Diabetes in Patients with Very High Risk

**Figure 5:**
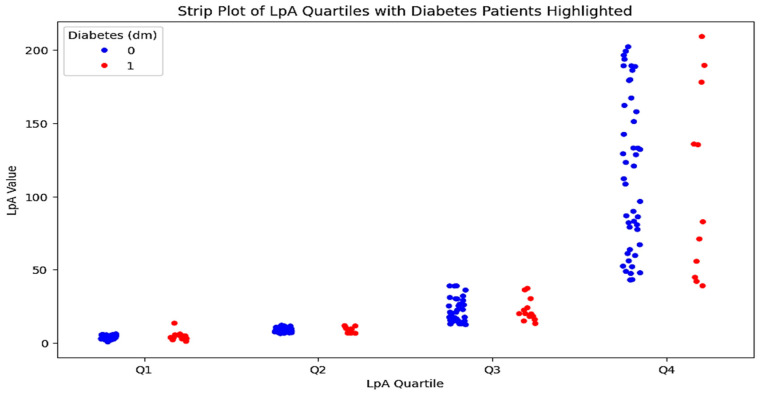
Quartile distribution of Lp(a) levels in patients with type 2 diabetes. The strip plot illustrates the quartile distribution of Lp(a) levels in patients with type 2 diabetes. The x-axis represents the Lp(a) quartiles (Q1–Q4), while the y-axis shows the Lp(a) values. Red dots indicate patients with type 2 diabetes, and blue dots represent patients without diabetes. The plot highlights the higher concentration of Lp(a) levels in the third and fourth quartiles for patients with type 2 diabetes.

To conduct a multivariate analysis and identify the most prognostically significant combination of factors, both groups were further divided into two subgroups each: those with diabetes/impaired glucose tolerance and those with a normal profile. The characteristics of patients in these subgroups are presented in Table 3. A detailed comparison of the indicators in patients with and without diabetes in the control group revealed no significant differences. However, an analysis of the data from the very high-risk group identified statistically significant differences in several indicators between the two subgroups. A graphical representation of these results is shown in Figure 5.

### 3.5. Multivariate Analysis

The next stage of this study involved conducting a multivariate analysis to identify the most significant predictors for the development of diabetes in patients with very high cardiovascular risk. This analysis sequentially included variables that showed statistically significant differences when comparing the control group with the very high-risk group, as well as within the subgroups based on the presence of diabetes. Adjusted odds ratios (ORs) were derived from multivariate logistic regression models including the following covariates: age, BMI, LDL-C, and ApoB. The results of the analysis are presented in Table 4A.

**Figure 6:**
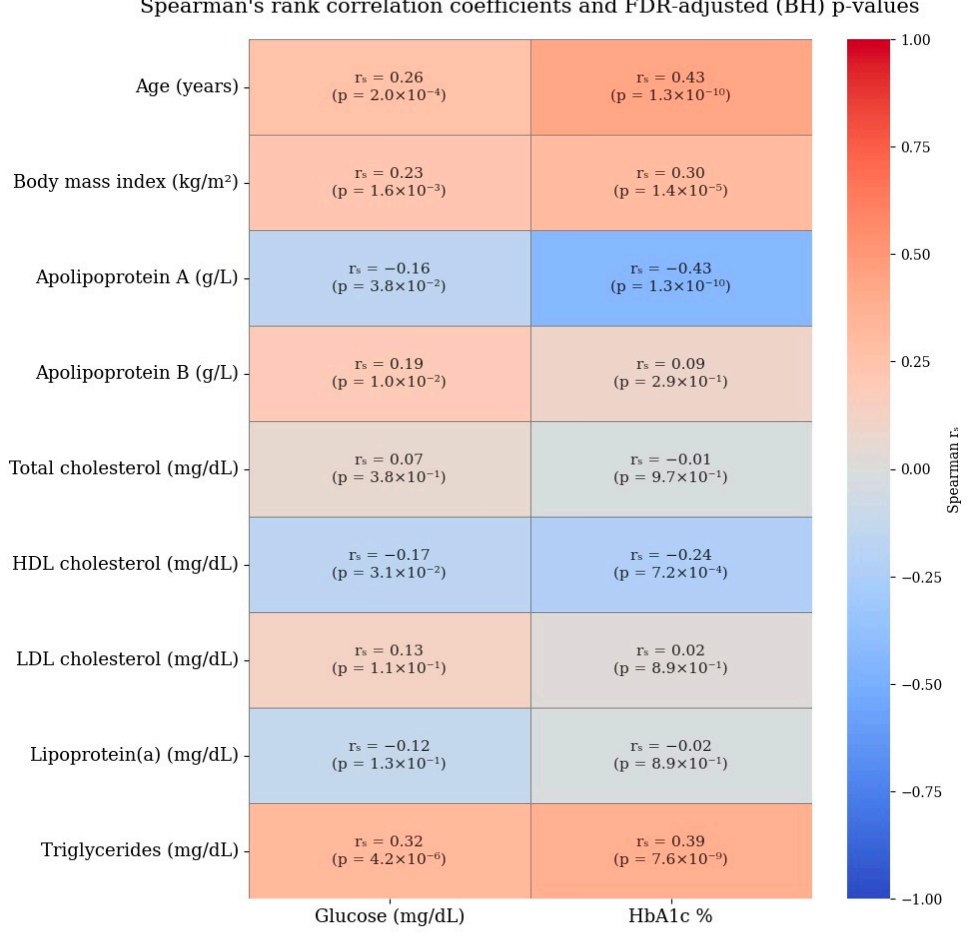
Spearman’s rank correlation coefficients for lipid and glycemic profiles.

Given the presence of a single extreme outlier value in the ApoB distribution (19 g/L) and the use of standardization, additional robustness analyses using two complementary approaches were conducted to evaluate the impact of this value on the magnitude and reliability of the ApoB–diabetes association. In the first robustness analysis approach, the outlier value was replaced with the sample median to minimize its influence on the standard deviation used for standard scaling. In the second approach, influential observations were identified using Cook’s distance (threshold: D > 4/n) and excluded from the analysis to account for high-leverage data points. Logistic regression models were re-estimated using the same covariates and standardized predictors, and the resulting effect estimates were compared to those from the original model. These analyses demonstrated that the association remained statistically significant, with the corresponding odds ratios stabilizing to more interpretable and consistent levels. Detailed results of these robustness analyses are presented in Table 4B. The Spearman’s rank correlation coefficients for lipid and glycemic profiles are provided in Figure 6.

## 4. Discussion

The exact mechanism underlying the relationship between lipoprotein(a) [Lp(a)] levels and the incidence of type 2 diabetes mellitus (T2DM) remains unclear. One hypothesis suggests that Lp(a) may contribute to insulin resistance, potentially through pro-inflammatory pathways or its influence on lipid metabolism [27]. Additionally, a complex interplay of genetic and non-genetic factors, including environmental influences and epigenetic modifications, may contribute to the ambiguous interaction between lipid profile components and diabetes risk [28]. It is also important to consider that T2DM develops gradually over time, indicating that the relationship between Lp(a) levels and glycemic profile indicators may evolve throughout the disease’s progression [29].

This study examined the association between lipid and glycemic profile parameters in patients with very high cardiovascular risk in Kazakhstan. A notable outcome of this study was the absence of a significant correlation between Lp(a) levels and glycemic markers. This observation aligns with some previous research while contrasting with studies that reported a negative correlation between Lp(a) and T2DM [30]. Our data showed consistently low Lp(a) levels among diabetic patients, challenging the hypothesis that elevated Lp(a) levels directly affect glucose metabolism. Unlike studies identifying low Lp(a) levels as a risk factor for T2DM, our results indicated no significant difference in Lp(a) distribution between diabetic and non-diabetic individuals [31].

This study investigated the relationship between lipid and glycemic parameters in a cohort of very high-cardiovascular-risk patients in Kazakhstan, comparing them to a low-risk control group. The key finding was the significant positive correlation between apolipoprotein B (Apo-B) levels and glycemic parameters, specifically fasting blood glucose (FBG: *r* = 0.40, *p* < 0.001) and glycated hemoglobin (HbA1c: *r* = 0.35, *p* = 0.001). This suggests that Apo-B is a potential predictor of type 2 diabetes mellitus (T2DM) in this population.

The observed association between Apo-B and glycemic dysregulation aligns with the existing literature in highlighting the role of Apo-B-containing lipoproteins in insulin resistance and impaired glucose metabolism. Elevated Apo-B levels reflect an increased concentration of atherogenic particles, which can promote inflammation, endothelial dysfunction, and impaired insulin signaling, contributing to the pathogenesis of T2DM. The lack of any statistically significant difference in the Apo-B levels between groups, despite their different levels of cardiovascular risk, suggests the key role of this parameter as a metabolic mediator rather than just a cardiovascular risk marker.

Triglycerides also showed moderate positive correlations with FBG (*r* = 0.29, *p* = 0.002) and HbA1c (*r* = 0.26, *p* = 0.009), reinforcing the established link between triglyceride metabolism and glucose regulation. Hypertriglyceridemia is often associated with insulin resistance and can further exacerbate metabolic dysfunction through various mechanisms, including increased hepatic glucose production and impaired insulin sensitivity.

In contrast, lipoprotein(a) [Lp(a)] levels exhibited no significant association with glycemic markers (*p* > 0.05), which contrasts with some previous studies that have suggested an inverse relationship between Lp(a) and T2DM. However, our finding is consistent with more recent data indicating a potential positive correlation between high Lp(a) levels and cardiovascular complications in diabetic patients. The lack of a direct correlation between Lp(a) and glycemic parameters in our study suggests that Lp(a) may primarily influence cardiovascular risk through mechanisms independent of glucose metabolism, such as by promoting inflammation, thrombosis, and atherosclerosis.

The weak negative correlations observed between apolipoprotein A (Apo-A) and glycemic parameters (FBG: *r* = −0.22, *p* = 0.01; HbA1c: *r* = −0.18, *p* = 0.04) suggest a potential protective role of HDL cholesterol in glucose metabolism. Higher Apo-A levels, reflecting increased HDL-C concentrations, have been associated with improved insulin sensitivity and reduced risk of T2DM in some studies.

These findings have several clinical implications. First, they highlight the importance of integrating lipid and glycemic assessments into cardiovascular risk management, particularly in high-risk populations [3]. Measuring Apo-B levels, in addition to traditional lipid parameters, may provide valuable insights into an individual’s risk of developing T2DM and could help guide targeted interventions aimed at improving both cardiovascular and metabolic outcomes. Second, the lack of an association between Lp(a) and glycemic parameters challenges the utility of Lp(a) as a primary marker of metabolic risk, suggesting that interventions targeting atherogenic lipid components, such as Apo-B and triglycerides, may be more effective in improving glycemic control and reducing cardiovascular events. Third, the potential protective effects of Apo-A underscore the importance of promoting lifestyle modifications and therapeutic strategies that raise HDL-C levels to improve insulin sensitivity and reduce metabolic risk.

This study also highlighted that Apo-B levels did not significantly differ between the very high-risk and low-risk groups, suggesting that while Apo-B is a predictor of diabetes within high-risk populations, it may not serve as a clear differentiating marker across broader cardiovascular risk categories. This observation implies that Apo-B’s role as a risk factor may be more critical in the context of existing metabolic disturbances rather than as a primary predictor of cardiovascular events in the general population. Additional research focusing on the interaction between Apo-B and other biomarkers of metabolic health could give a more nuanced understanding of its clinical utility.

Our study provides valuable insights into the complex interplay between lipid metabolism and glycemic control in high-cardiovascular-risk populations. By identifying Apo-B and triglycerides as key predictors of T2DM and clarifying the role of Lp(a) in metabolic risk stratification, our findings can inform clinical decision-making and guide future research aimed at improving both cardiovascular and metabolic outcomes. Future studies should focus on longitudinal investigations to assess the causal relationships between lipid and glycemic parameters and to evaluate the effectiveness of targeted interventions in reducing the risk of T2DM and cardiovascular events in high-risk individuals [32,33]. Lower Apo-A levels in diabetic patients may reflect impaired HDL function, reduced reverse cholesterol transport, and a diminished protective effect against oxidative stress and vascular inflammation. These findings are consistent with previous studies suggesting that maintaining optimal Apo-A levels could contribute to better glycemic control and lower cardiovascular risk [34,35]. Exploring the therapeutic potential of HDL-raising strategies, including lifestyle modifications and pharmacological agents, might offer additional benefits for patients with combined dyslipidemia and glucose intolerance.

## 5. Study Limitations and Strengths

The cross-sectional design of this study represents a limitation, as it restricts its ability to establish causality between lipid and glycemic parameters. The relatively small sample size may also affect the statistical power to detect subtle interactions. However, this study’s strength lies in its focus on a very high- cardiovascular-risk population in Kazakhstan, providing valuable insights into a demographic that has been underrepresented in prior research. Future studies should aim to validate these findings through longitudinal research with larger, more diverse cohorts and explore the mechanistic pathways linking lipid metabolism to glucose homeostasis.

## 6. Conclusions

This study provides important insights into the complex relationship between lipid and glycemic profile parameters in patients with a very high cardiovascular risk in Kazakhstan. The findings revealed that while traditional lipid markers such as triglycerides and apolipoprotein B (Apo-B) were positively associated with glycemic parameters, no significant correlation was observed between lipoprotein(a) [Lp(a)] levels and indicators of glucose metabolism. These results contribute to the growing body of evidence that Lp(a) is a recognized risk factor for atherosclerotic cardiovascular diseases, but its role in type 2 diabetes mellitus (T2DM) development remains ambiguous.

Apo-B emerged as a significant predictor of T2DM in the high-risk population, highlighting its potential as a biomarker for cardiovascular risk assessment and identifying individuals at increased risk of metabolic disorders. The observed correlations between triglyceride levels and glycemic markers further underscore the interplay between lipid metabolism and glucose homeostasis, suggesting that managing hypertriglyceridemia could contribute to better glycemic control and reduce cardiovascular risk.

The absence of a clear relationship between Lp(a) levels and glycemic parameters aligns with previous studies suggesting that the influence of Lp(a) on glucose metabolism may be indirect or moderated by other factors, including genetic predisposition and environmental influences. This finding emphasizes the need for further research to elucidate the potential mechanisms underlying this relationship, particularly in diverse populations with unique genetic and lifestyle characteristics such as those in Kazakhstan.

These findings underscore the importance of a multifaceted approach to cardiovascular risk management that integrates both lipid and glycemic assessments. Such an approach can enhance early risk detection and support personalized treatment strategies, ultimately improving clinical outcomes in very high-risk patients. Future studies should focus on longitudinal analyses and explore the potential impact of targeted lipid-lowering therapies on glycemic control, contributing to the development of more effective strategies for managing cardiovascular and metabolic diseases in this vulnerable population.

## Figures and Tables

**Table 1:** Lipid profile values for low-risk and very high-risk groups associated with atherosclerotic cardiovascular disease.

Lipid Profile	Low-Risk Group	Very High-Risk Group
LDL Cholesterol (LDL-C)	<3.0 mmol/L (<100 mg/dL)	>4.9 mmol/L (<190 mg/dL)
Non-HDL Cholesterol	<3.4 mmol/L (<131 mg/dL)	Not specifically stated; inferred similar to LDL
Apolipoprotein B (Apo-B)	<90 mg/dL	≥140 mg/dL
High-Density Lipoprotein (HDL-C)	No specific value; inversely related to risk	No specific value; inversely related to risk
Lipoprotein(a) [Lp(a)]	<30 mg/dL	≥50 mg/dL
Total Cholesterol	<200 mg/dL	>310 mg/dL

**Table 2:** Glycemic profile values for low-risk and very high-risk groups.

Glycemic Profile	Low-Risk Group	High-Risk Group
Fasting Blood Glucose	≤5.6 mmol/L (≤100 mg/dL)	≥7.0 mmol/L (≥126 mg/dL)
Glycated Hemoglobin (HbA1c)	≤5.6% (≤38 mmol/mol)	≥6.5% (≥48 mmol/mol)

**Table 3:** Baseline characteristics of study participants.

Parameter	Study Group (*n* = 151)	Control Group (*n* = 73)	Statistic
**Age (in years)**	58 ± 6.7	48 ± 10.4	U = 2395.5 *p* = 1.72 × 10^−12^
**Gender (men)**	70.8%	40.8%	χ^2^ = 15.118 *p* = 0.00010
**BMI (kg/m^2^)**	29.63 ± 5.11	27.46 ± 4.79	U = 3968.5 *p* = 0.00067
**Total cholesterol (mmol/L)**	197.39 ± 34.41	190.15 ± 49.67	U = 6441.5 *p* = 0.0408
**Triglycerides (mmol/L)**	155.18 ± 80.02	120.94 ± 76.17	U = 3635.5 *p* = 3.5131 × 10^−5^
**TC—LDL (mmol/L)**	127.76 ± 43.52	130.19 ± 30.04	U = 6085.0 *p* = 0.2078
**OX—HDL (mmol/L)**	45.52 ± 11.74	55.60 ± 14.60	U = 8110.0 *p* = 1.04401 × 10^−8^
**Apo-A (g/L)**	1.12 ± 0.70	1.44 ± 0.26	U = 9425.5 *p* = 6.4003 × 10^−18^
**Fasting glucose level (mmol/L)**	124.09 ± 53.35	100.94 ± 9.04	U = 3605.5 *p* = 2.6269 × 10^−5^
**HbA1c (%)**	6.50 ± 1.75	5.02 ± 0.49	U = 1180.0 *p* = 1.1989 × 10^−21^
**Presence of diabetes mellitus**	31 (20.5%)	2 (2.8%)	χ^2^ = 26.483 *p* =2.6584 × 10^−7^
**Impaired glucose tolerance**	18 (11.9%)	1 (1.4%)	χ^2^ = 11.590 *p* = 0.0007
**Height cm**	165.84 ± 8.56	168.9 ± 7.37	U = 4603.0 *p* = 0.0448
**Weight**	83.68 ± 13.43	75.75 ± 15.56	U = 3572.0 *p* = 1.8781 × 10^−5^

**Table 4:** (A) Multivariate analysis of identifying predictors of type 2 diabetes in patients at high risk. (B) Robustness analyses of the ApoB–diabetes association.

**(A)**						
**Variables,** **(Mean ± SD)**	**Very High-Risk Group** **(*n* = 151)**					
	**Crude OR**	**95% CI**	***p*-Value**	**Adjusted OR**	**95% CI**	***p*-Value**
**Age**	1.046	[0.991; 1.104]	0.105	1.3685	[0.929, 2.0138]	0.111
**BMI, kg/m^2^**	1.048	[0.981; 1.12]	0.167	1.1669	[0.818, 1.6630]	0.393
**LDL, (mg/dL)**	1.006	[0.998; 1.014]	0.116	0.5176	[0.2247, 1.1921]	0.122
**Apo B, (g/L)**	4.413	[1.472; 13.229]	0.008	141.3983	[2.834, 7053.91]	0.013
**(B)**						
**Model description**			**Adjusted OR (ApoB)**	**95% CI**		***p*-value**
Original model using standardized predictors with extreme ApoB value retained (19 g/L)			141.4	[2.83–7053.91]		0.013
Outlier-corrected model: extreme ApoB value (>10 g/L) replaced with sample median prior to standardization			2.97	[1.27–6.94]		0.012
Outlier-corrected model with influential observations removed (identified by Cook’s distance, D > 4/n) using standardized predictors			7.50	[2.31–24.28]		0.001

Adjusted odds ratios (ORs) were derived from multivariate logistic regression models including the following covariates: age, BMI, LDL-C, and ApoB.

## Data Availability

The raw data supporting the conclusions of this article will be made available by the authors on request.
